# Molecular ontogeny underlies the benefit of adding venetoclax to hypomethylating agents in newly diagnosed AML patients

**DOI:** 10.1038/s41375-024-02230-w

**Published:** 2024-03-27

**Authors:** Shai Shimony, Jacqueline S. Garcia, Julia Keating, Evan C. Chen, Marlise R. Luskin, Maximilian Stahl, Donna S. Neuberg, Daniel J. DeAngelo, Richard M. Stone, R. Coleman Lindsley

**Affiliations:** 1 Division of Hematologic Neoplasia, Department of Medical Oncology, Dana-Farber Cancer Institute, Boston, MA USA; 2 Rabin Medical Center and Faculty of Medicine, Tel Aviv University, Tel-Aviv, Israel; 3 Department of Data Science, Dana-Farber Cancer Institute, Boston, MA USA

**Keywords:** Acute myeloid leukaemia, Targeted therapies

## Abstract

The clinical impact of molecular ontogeny in acute myeloid leukemia (AML) was defined in patients treated with intensive chemotherapy. In a cohort of 314 newly diagnosed AML patients, we evaluated whether molecular ontogeny subgroups have differential benefit of venetoclax (VEN) added to hypomethylating agents (HMA). In secondary ontogeny (*n* = 115), median overall survival (OS)(14.1 *vs*. 6.9 months, *P* = 0.0054), composite complete remission (cCR 61% *vs*. 18%, *P* < 0.001) and allogeneic hematopoietic stem cell transplant (alloHCT) (24% *vs*. 6%, *P* = 0.02) rates were better in patients treated with HMA + VEN *vs*. HMA. In contrast, in *TP53* AML(*n* = 111) median OS (5.7 *vs*. 6.1, *P* = 0.93), cCR (33% vs. 37%, *P* = 0.82) and alloHCT rates (15% *vs*. 8%, *P* = 0.38) did not differ between HMA + VEN vs. HMA. The benefit of VEN addition in the secondary group was preserved after adjustment for significant clinicopathologic variables (HR 0.59 [95% CI 0.38–0.94], *P* = 0.025). The OS benefit of HMA + VEN in secondary ontogeny was similar in those with *vs*. without splicing mutations (*P* = 0.92). Secondary ontogeny AML highlights a group of patients whose disease is selectively responsive to VEN added to HMA and that the addition of VEN has no clinical benefit in *TP53*-mutated AML.

## Introduction

In patients with newly diagnosed acute myeloid leukemia (AML) specific somatic mutations are associated with prior myeloid disease [1]. Mutations that define “secondary ontogeny” are now integrated into AML diagnostic criteria as myelodysplasia-related defining mutations [2, 3]. Based on data from patients treated initially with intensive cytotoxic induction including typically an anthracycline and cytarabine, secondary ontogeny mutations are associated with worse survival and thus confer adverse risk assignment per the 2022 European Leukemia Network (ELN) risk schema [1, 4, 5]. However, whether secondary mutations confer a worse prognosis in patients treated with hypomethylating agents (HMA) with or without venetoclax (VEN) is unknown and the relevance of ELN 2022 criteria in these patients is unclear.

Post-hoc analyses of the VIALE-A trial [6] showed that the incremental benefit of adding VEN to HMA-based chemotherapy was not uniform and varied based on the presence of distinct mutations, such as *FLT3* [7], *IDH1/2* [8] and *TP53* [7, 9]. However, as treatment with HMA + VEN has expanded beyond the traditional label and is commonly used in patients aged less than 75 who are potential transplant candidates or candidates for more intensive chemotherapy, The effect of mutational pattern on outcomes may differ from the post-hoc analyses of the VIALE-A. Given the financial cost and associated toxicity (cytopenias, infection) [6] with the addition of VEN to HMA chemotherapy, it is important to identify which patients benefit from the addition of VEN.

Here, we evaluated whether secondary molecular ontogeny implies adverse risk in AML patients treated with HMA + VEN and assessed the benefit of adding VEN to HMA by molecular ontogeny group.

## Methods

### Patients

We retrospectively identified consecutive patients with newly diagnosed AML at Dana-Farber Cancer Institute (DFCI) between August 2014 and July 2022 who were treated with HMA-based therapy, defined as either HMA (inclusive of HMA monotherapy or HMA + non-VEN drug) or HMA + VEN (treated with HMA + VEN). The presence of pathogenic mutations was assessed by next-generation sequencing, as previously described [10]. We excluded patients who had acute promyelocytic leukemia and those who received no treatment or other treatment. Ontogeny as de novo, secondary, or *TP53* was classified by previously defined hierarchy [1], (Supplementary Fig. 1A): patients with *TP53* mutations were assigned “*TP53* group”, patients without *TP53* but with one or more secondary ontogeny defining mutations (*ASXL1*, *BCOR*, *EZH2*, *SF3B1*, *SRSF2*, *STAG2*, *U2AF1*, and *ZRSR2*) comprise the “secondary group”, and the remaining patients defined as “de novo group”. We divided the *TP53* group into subgroups by variant allele frequency (VAF) cut-offs (10%, 20%, and 50%) to determine if the potential benefit of VEN addition differs between these subgroups. We further divided the secondary group into “splicing subgroup” (those with at least one splicing mutation: *SF3B1*, *SRSF2*, *U2AF1*, or *ZRSR2*) or “non-splicing subgroup” (without any splicing mutation). Consistent with the WHO 5^th^ edition [3] and based on our hierarchical model [1], we did not include *RUNX1* in the secondary ontogeny group, since it is not highly specific for secondary AML (with a cut-off of 95% specificity) and is thus not ontogeny-defining. In the international consensus classification (ICC) criteria [2] *RUNX1* was grouped with secondary ontogeny-specific mutations (to compose the chromatin/RNA-splicing group), based on a Bayesian analysis defining 11 genomic AML subgroups [11]. We performed a separate analysis with *RUNX1* as a secondary ontogeny defining mutation to enable cross-classification comparison, and conducted a sensitivity analysis with *RUNX1* as a secondary ontogeny defining mutation to determine whether results would differ with alternative classification.

### Outcomes

Responses were defined by the ELN 2022 response criteria [1, 4, 5]; for each patient, we included the best response documented. The composite complete remission (cCR) rate was calculated as complete remission (CR) plus complete remission with incomplete count recovery (CRi). Overall survival (OS) was calculated from the date of first treatment until death or last follow-up.

### Statistics

Categorical variables were summarized as counts and percentages, and comparisons were made by Fisher’s exact test. Continuous variables were summarized as median and range or interquartile range (IQR), and comparisons were made by Wilcoxon rank-sum or Kruskal–Wallis tests. Correction for multiple comparisons was conducted by the Holm–Bonferroni method. Overall survival was estimated by the Kaplan-Meier method and the log-rank test was used to compare survival outcomes. Cox proportional hazards regression models were fitted to assess the effect of covariates with allogeneic hematopoietic cell transplant (alloHCT) as a time-varying covariate, both in the entire group and within each ontogeny group. All covariates that were found to be significant in the univariable analyses with a *p* < 0.1, as well as pre-defined covariates (ontogeny group, treatment [HMA + VEN *vs*. HMA] and alloHCT) were candidates for the multivariable analysis. The final model was fitted in a backward stepwise fashion. For all analyses, the confidence interval (CI) was calculated at the (two-sided) 95% confidence level. A two-sided *p*-value of <0.05 was considered statistically significant. All statistics were performed using R version 4.2.2.

## Results

### Patients

**Fig. 1:**
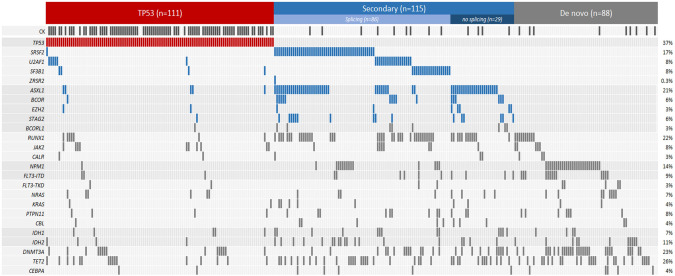
Cytogenetic and molecular plot by groups. CK complex karyotype.

Overall, 314 patients with newly diagnosed AML were included in the analysis. The median age was 74 years (range 25–90) and 111 (35%) had antecedent myeloid neoplasm. Most patients (75%) were classified as adverse risk by ELN 2022. In total,166 (53%) were treated with HMA + VEN, and 148 (47%) were treated with HMA (127 [40%] with HMA monotherapy and 21 [7%] with HMA + non-VEN drug), Table 1. There were no significant differences in baseline characteristics between patients treated with HMA *vs*. HMA + VEN. When classified by ontogeny, 111 (35%) were in the *TP53* group (60 with HMA + VEN, 51 with HMA); 115 (37%) in the secondary ontogeny group (68 with HMA + VEN, 47 with HMA); and 88 (28%) in the de novo ontogeny group (38 with HMA + VEN, 50 with HMA, Fig. 1 and Supplementary Fig. 1B). Decitabine was more commonly used compared with azacytidine (*n* = 208 [66%] vs. *n* = 106 [34%)], with five days of decitabine being the most common HMA regimen used (*n* = 166 [53%], Supplementary Table 1).

**Table 1:** Patient characteristics by treatment.

	Overall (*N* = 314)	Treatment group, *N* (%)		*p*-value
		HMA^a^ (*N* = 148)	HMA + VEN (*N* = 166)	
Sex (male)	186 (59)	91 (61)	95 (57)	0.5
Age (years, median, range)	73.7 (25.2, 89.6)	74.0 (42.4, 88.5)	73.7 (25.2, 89.6)	0.4
Age categories (% within age group)				
– <60 years	32 (10)	11 (7)	21 (13)	0.4
– 60 to <75 years	152 (48)	72 (49)	80 (48)	>0.9
– ≥75 years	130 (41)	65 (44)	65 (39)	0.8
Prior MDS, MPN or MDS/MPN overlap	111 (35)	48 (32)	63 (38)	0.3
Prior myeloid disease directed therapy				
– HMA	35 (11)	17 (11)	18 (11)	0.9
– Prior alloHCT	29 (9)	12 (8)	17 (10)	0.6
– t-AML	87 (28)	39 (26)	48 (29)	0.7
Cytogenetics^b^				
– Normal	80 (29)	38 (29)	42 (29)	0.9
– 5/7/17 abnormalities	94 (34)	47 (36)	47 (32)	0.4
– Complex karyotype	116 (42)	55 (43)	61 (41)	>0.9
ELN 2022 risk stratification				
– Favorable	39 (12)	15 (10)	24 (14)	>0.9
– Intermediate	40 (13)	21 (14)	19 (11)	>0.9
– Adverse	235 (75)	112 (76)	123 (74)	0.8
Ontogeny group				
– De-novo	88 (28)	50 (34)	38 (23)	0.1
– Secondary	115 (37)	47 (32)	68 (41)	0.2
– TP53	111 (35)	51 (34)	60 (36)	0.8

*HMA* hypomethylating agents, *VEN* venetoclax, *MDS* myelodysplastic syndrome, *MPN* myeloproliferative syndrome, *alloHCT* allogeneic hematopoietic stem cell transplantation, *t-AML* - therapy related AML, *CBF* core binding factor, *ELN* European leukemia network, *AML* acute myeloid leukemia, *Ven* venetoclax.

^a^21/148 (14%) in the HMA group received additional non-venetoclax drug: FLT3 inhibitors (*n* = 5), CD33 antibody (*n* = 5),APR246 (*n* = 3), Syk inhibitor (*n* = 2), ipilimumab (*n* = 2) and one of each of the following – CD123 antibody, CD47 antibody, IDH1 inhibitor, MUC1 inhibitor.

^b^Cytogenetics known for 77 De-novo patients, 102 Secondary patients and 103 TP53 patients.

### Survival outcomes

#### Survival in the entire group

**Fig. 2:**
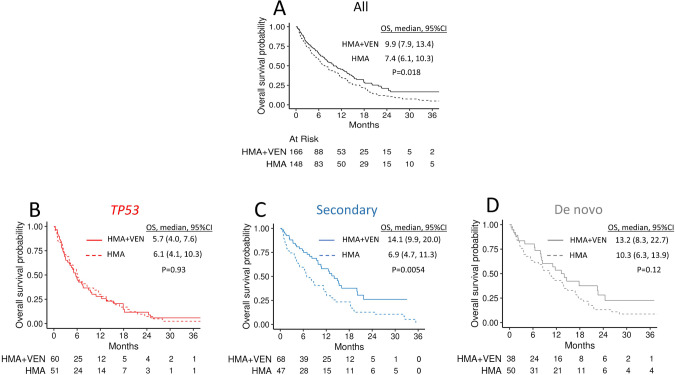
Overall survival of the entire cohort and in each ontogeny. **A** All patients. **B** *TP53* ontogeny group. **C** Secondary ontogeny group. **D** De novo ontogeny group. OS overall survival, CI confidence interval, HMA hypomethylating agents, VEN venetoclax.

After a median follow-up of 29 months (95% CI 24–32), the OS was better in patients treated with HMA + VEN compared with those who received HMA without VEN (median OS 9.9 months [95% CI 7.9–13.4] *vs*. 7.4 months [95% CI 6.1–10.3], respectively, *P* = 0.018, Fig. 2A). The results were similar in a sensitivity analysis excluding patients who received HMA+ an additional non-VEN drug (*n* = 21, Supplementary Fig. 2). In a multivariable analysis, only *TP53* ontogeny (HR 1.88 [95% CI 1.37–2.59]) and consolidation with alloHCT (HR 0.23 [95% CI 0.14–0.40]) were independently associated with OS (Supplementary Table 2).

#### Survival in each ontogeny group

To define the ontogeny-specific activity of VEN, we performed a separate analysis in each molecularly defined ontogeny group. Among patients with *TP53* mutated disease, there was no difference in OS between HMA + VEN *vs*. HMA treatment groups (5.7 months [95% CI 4–7.6] *vs*. 6.1 months [95% CI 4.1–10.3], *P* = 0.93), Fig. 2B. Furthermore, OS was comparable in both treatment groups among patients with *TP53* mutations irrespective of VAF cutoff, including 10%, 20%, and 50% (Supplementary Table 3). In the de novo group, OS was not different between HMA + VEN *vs*. HMA (13.2 months [95% CI 8.3–22.7] *vs*. 10.3 months [95% CI 6.3–13.9], *P* = 0.12, Fig. 2C). Conversely, in the secondary group, patients treated with HMA + VEN had significantly better OS compared to patients treated with HMA (14.1 months [9.9–20.0] *vs*. 6.9 months [95% CI 4.7–11.3], *P* = 0.0054, Fig. 2D). The results were retained in a sensitivity analysis when *RUNX1* was considered as a secondary ontogeny-defining mutation (Supplementary Fig. 3).

To identify ontogeny-specific prognostic variables, we conducted Cox multivariable regression models within each ontogeny group separately. Across all groups, alloHCT was associated with improved OS (de novo: HR 0.14 [95% CI 0.03–0.58], *P* = 0.0067; secondary: HR 0.40 [95% CI 0.18–0.89], *P* = 0.026; *TP53*: HR 0.17 [95% CI 0.07–0.42], *p* < 0.001). Conversely, treatment modality (HMA + VEN *vs*. HMA) was associated with improved OS only in the secondary group (de novo: HR 0.67 [95% CI 0.40–1.12], *P* = 0.13; secondary: HR 0.59 [95% CI 0.38–0.94], *P* = 0.025; *TP53*: HR 1.19 [95% CI 0.78–1.81], *P* = 0.42).

#### Sensitivity analysis without TP53 group

Since *TP53* mutations were strongly associated with worse outcomes and were highly prevalent in the cohort, their inclusion in the analysis could obscure a meaningful effect of initial treatment on survival in patients without *TP53* mutations. We conducted a sensitivity analysis and regression modeling for OS in patients without *TP53* mutations (*n* = 203). Among these patients, the median OS was 14.0 months (95% CI 10.6–17.8) with HMA + VEN *vs*. 8.7 months (95% CI 6.3–11.4) with HMA, *P* = 0.0028 (Supplementary Fig. 4). In the multivariable analysis, both alloHCT (HR 0.27 [95% CI 0.13–0.53, *p* < 0.001]) and treatment with HMA + VEN *(vs*. HMA) (HR 0.66 [95% CI 0.47–0.92], *P* = 0.015) were associated with improved OS (Supplementary Table 4).

#### Survival in patients treated with HMA + VEN

**Fig. 3:**
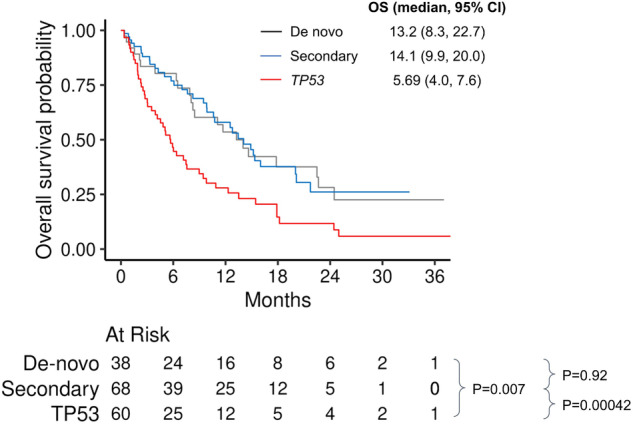
Overall survival by ontogeny in patients treated with HMA + VEN. CI confidence interval, HMA hypomethylating agents, VEN venetoclax.

In patients treated with HMA + VEN the OS was similar between de novo and secondary groups (median OS 13.2 months [95% CI 8.3–22.7] *vs*. 14.1 months [95% CI 9.9–20.0], respectively, *P* = 0.92); and each group had better OS compared to *TP53* group (median 5.7 months [95% CI 4.0–7.6], comparison *vs*. de novo *P* = 0.007; comparison *vs*. secondary *p* < 0.001), Fig. 3. In the multivariable analysis of patients treated with HMA + VEN, the comparable OS between secondary and de novo groups was retained (HR 1.07 [95% CI 0.63–1.84, *P* = 0.79]), as well as the worse survival in the *TP53* group (compared with de novo, HR 2.57 [95% CI 1.53–4.33, *P* < 0.001]) and better survival with alloHCT as a time-varying covariate (HR0.22 [95% CI 0.11–0.46], *P* < 0.001, Table 2).

**Table 2:** Univariable and multivariable Cox regression overall OS of patients treated with HMA + VEN.

Covariate	Univariable analysis (HR, CI 95%)	*p*-value	Multivariable analysis (HR, CI 95%)	*p*-value
Age	1.01 (0.99, 1.02)	0.61		
Sex (relative to female)	0.61 (0.41, 0.90)	0.013		
Prior myeloid disease	1.12 (0.75, 1.65)	0.59		
Prior HMA exposure	1.22 (0.68, 2.18)	0.50		
Molecular				
– Ontogeny group (relative to De-novo)				
○Secondary	0.95 (0.55, 1.62)	0.84	1.07 (0.63, 1.84)	0.79
○*P53*	2.06 (1.24, 3.44)	0.0055	2.57 (1.53, 4.33)	<0.001 (3.9e−4)
– *FLT3-ITD*	1.22 (0.65, 2.29)	0.54		
– *NPM1*	0.92 (0.50, 1.67)	0.77		
– *IDH1*	0.77 (0.36, 1.66)	0.50		
– *IDH2*	0.94 (0.48, 1.87)	0.87		
– *NRAS/KRAS*	1.20 (0.65, 2.20)	0.56		
– *JAK2/CALR*	1.42 (0.82, 2.46)	0.21		
Cytogenetics (relative to normal)^a^				
– Complex	2.18 (1.26, 3.76)	0.0053		
– Others	1.19 (0.64, 2.19)	0.58		
Treatment				
– alloHCT as time-varying covariate	0.27 (0.13, 0.54)	<0.001 (2.6e−4)	0.22 (0.11, 0.46)	<0.001 (4.2e−5)

*OS* overall survival, *HMA* hypomethylating agent, *VEN* venetoclax, *HR* hazard ratio, *CI* confidence interval, *FLT3-ITD* fms-like tyrosine kinase 3 internal tandem duplication, *NPM1* nucleophosmin 1, *IDH* isocitrate dehydrogenase, *NRAS* neuroblastoma rat sarcoma, *KRAS* kirsten rat sarcoma, *JAK2* janus kinase 2, *CALR* calreticulin, *alloHCT* allogeneic hematopoietic stem cell transplantation.

^a^19 patients were omitted from the univariable regression analysis for cytogenetics due to missing data.

### Response and transplant rates

To determine whether the distinct survival patterns in each ontogeny were associated with other measures of clinical response, we evaluated cCR and alloHCT rates in the entire cohort and within each ontogeny group separately. Overall, the cCR rate was better in patients treated with HMA + VEN than in those who received HMA (49% *vs*. 28%, *P* = 0.001). When analyzing molecularly defined ontogeny groups separately, the cCR rates were higher in patients treated with HMA + VEN *vs*. HMA in the de novo group (54% *vs*. 29%, *P* = 0.034]) and secondary group (61% *vs*.18%, *p* < 0.001), but not different in patients with *TP53* mutations (33% *vs*. 37%, *P* = 0.82, Table 3).

**Table 3:** Best response and alloHCT by ontogeny.

	Ontogeny group, *N* (%)								
	De novo			Secondary			*TP53*		
	*HMA*^a^ *(N* = *50)*	*HMA* + *VEN (N* = *38)*	*p*-value	*HMA*^a^ *(N* = *47)*	*HMA* + *VEN (N* = *68)*	*p*-value	*HMA*^a^ *(N* = *51)*	*HMA* + *VEN (N* = *60)*	*p*-value
Best response	11 (29)	19 (54)	0.034	6 (18)	34 (61)	<0.001	13 (37)	18 (33)	0.82
cCR (CR+CRi)									
CR	8 (21)	10 (29)	0.59	2 (6)	13 (23)	0.041	9 (26)	10 (19)	0.44
Cri	3 (8)	9 (26)	0.058	4 (12)	21 (38)	0.0084	4 (11)	8 (15)	0.76
MLFS	1 (3)	3 (9)	0.34	2 (6)	9 (16)	0.2	4 (11)	8 (15)	0.76
PD	26 (68)	13 (37)	0.01	26 (76)	13 (23)	<0.001	18 (51)	28 (52)	>0.99
Missing	12	3	–	13	12	–	16	6	–
AlloHCT	5 (10)	4 (11)	>0.99	3 (6)	16 (24)	0.020	4 (8)	9 (15)	0.38

*HMA* hypomethylating agents, *VEN* venetoclax, *cCR* composite complete remission, *CR* complete remission, *CRi* complete remission with incomplete count recovery, *MLFS* morphologic leukemia-free state, *PD* progressive disease, *alloHCT* allogeneic hematopoietic stem cell transplantation.

^a^21/148 (14%) in the HMA group received additional non-venetoclax drug: FLT3 inhibitors (*n* = 5), CD33 antibody (*n* = 5),APR246 (*n* = 3), Syk inhibitor (*n* = 2), ipilimumab (*n* = 2) and one of each of the following – CD123 antibody, CD47 antibody, IDH1 inhibitor, MUC1 inhibitor.

As long-term survival among patients with high-risk AML subtypes depends on alloHCT [12], we evaluated whether HMA + VEN may be associated with higher alloHCT rates in the entire cohort and in each ontogeny group. Overall, 41 (13%) patients were consolidated with alloHCT, with patients treated with HMA + VEN more frequently transplanted (17%, *n* = 29) than those treated with HMA (8%, *n* = 12), *P* = 0.018. However, when analyzed within each ontogeny group, the rates of alloHCT were similar within the *TP53* group (15% [*n* = 9] *vs*. 8% [*n* = 4], *P* = 0.38) and in the de novo group (11% [*n* = 4] *vs*.10% [*n* = 5], *p* > 0.99). Conversely, the rates were higher among patients in the secondary group treated with HMA + VEN (24%, *n* = 16) *vs*. HMA (6%, *n* = 3), *P* = 0.02.

### Subgroup analysis of secondary ontogeny

**Fig. 4:**
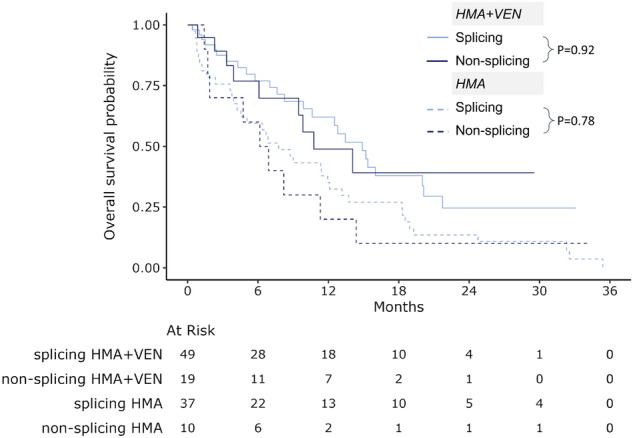
Overall survival in secondary ontogeny by treatment and splicing mutations. CI confidence interval, HMA hypomethylating agents, VEN venetoclax.

Mutations that affect RNA splicing (*SF3B1*, *SRSF2*, *U2AF1*, *ZRSR2*) have been reported to be associated with improved survival in patients treated with VEN-based regimens[13]. To determine whether the aforementioned response and survival benefit with HMA + VEN *vs*. HMA in the secondary ontogeny group is attributable solely to splicing abnormalities in these mutations, we compared patients with (*n* = 86) *vs*. without (*n* = 29) these mutations within the secondary group. First, splicing *vs*. non-splicing subgroups were comparable in age, prior therapy, or myeloid malignancy rates and ELN2022 risk criteria (Supplementary Table 5). The splicing *vs*. non-splicing subgroup had higher rates of *NPM1* (16% *vs*. 0%, respectively *P* = 0.02) and lower rates of *ASXL1* (42% *vs*. 79%, respectively, *P* < 0.001). The cCR rates between splicing and non-splicing mutation subgroups were comparable among patients treated with HMA (22 vs. 0%, *P* = 0.3) or HMA + VEN (61% VS. 60%, *P* > 0.99). Similarly, OS was comparable between splicing and non-splicing subgroups treated with HMA (median OS 7.8 months [95% CI 4.2–12.1] *vs*. 6.5 months [95% CI 1.9-11.3], *P* = 0.78) or with HMA + VEN (median OS 14.9 months [95% CI 10.6–20.1] *vs*. 10.8 months [95% CI 6.1-NR], *P* = 0.92), Fig. 4.

## Discussion

Molecular ontogeny defines three AML subgroups (*TP53*, secondary, and de novo), each with different clinicopathologic characteristics and distinct response and survival outcomes in patients receiving intensive induction therapy [1, 4, 5]. It remains unclear whether a specific molecular ontogeny is also associated with distinct clinical outcomes in patients treated with HMA + VEN or if the benefit of adding VEN to HMA is similar across all molecular ontogeny groups.

In this study, we found that in newly diagnosed patients with AML, treatment with HMA + VEN compared with HMA had a significant benefit among those with secondary ontogeny mutations. Patients with secondary mutations treated with HMA + VEN had prolonged OS, better response, and increased alloHCT rates compared with HMA alone, thereby achieving an OS similar to de novo AML. In stark contrast, patients with *TP53* mutation had similarly dismal outcomes when treated with HMA with or without VEN. Their outcomes were indistinguishable, suggesting that VEN only added cost and toxicity. Our findings support a re-evaluation of secondary ontogeny mutations as an adverse risk in patients treated with HMA + VEN and challenge the benefit of adding VEN to HMA chemotherapy in patients with *TP53* mutations.

In secondary ontogeny, VEN added to HMA specifically overcomes the dismal outcome in secondary ontogeny patients treated with HMA (which was as poor as that of the *TP53* ontogeny), doubles the median OS (6.9 to 14.1 months), and equalizes it to de novo patients treated with HMA + VEN (14.1 *vs*. 13.2 months, respectively, *P* = 0.92). Although the mechanism for the marked improvement in secondary ontogeny OS is unknown, it may relate, in part, to pre-clinical and clinical studies demonstrating the sensitivity of splicing mutations to BCL2 inhibition [14, 15]. A recent study demonstrated that VEN addition to low dose chemotherapy abrogates the adverse risk of splicing mutations [13]. Our analysis showed improved OS across all secondary ontogeny AML, irrespective of splicing factor mutations, suggesting that additional mechanisms may contribute to this survival advantage.

We did not find any significant improvement in survival, responses, or rates of alloHCT in patients with *TP53* mutated AML treated with HMA + VEN *vs*. HMA. The poor OS and lack of improvement with the addition of VEN were consistent across patients with *TP53* mutations, irrespective of VAF. This is consistent with a previous study demonstrating high *TP53* VAF (>40%) was associated with worse OS only among patients with *TP53* mutated AML treated with intensive chemotherapy, but not in those treated with HMA-based therapy [16]. Overall, these findings are similar to previous studies showing dismal prognosis in *TP53* mutated AML irrespective of initial treatment [17], with long-term survival achieved only in patients who were consolidated with alloHCT [18–20]. As the addition of VEN to HMA is associated with prolonged cytopenias [21, 22] and more frequent infections [21], the addition of VEN in *TP53* mutated AML is questionable and novel strategies should be pursued in this challenging to-treat population.

Previous studies have reported differential activity of VEN in specific molecular subgroups within de novo AML, including a benefit in AML with *NPM1* mutations or a detriment in AML with FLT3-ITD [6, 23]. Taken as a whole, we observed no statistically significant impact of VEN in the de novo ontogeny group, which may reflect the molecular heterogeneity of this subgroup combined with our modest cohort size, or the higher overall efficacy of HMA monotherapy in de novo patients.

Prognostic scoring systems, such as ELN, link disease and patient characteristics with clinical outcomes after specific standard-of-care therapies. We found that among patients treated with HMA + VEN, the OS was similar between the de novo (median OS 13.2 months) *vs*. secondary (median OS 14.1 months) ontogenies and better than *TP53* ontogeny (median OS 5.7 months). This effect was driven by the particular benefit of VEN in secondary ontogeny and no improvement in *TP53-mutated* AML. Our findings of the differential benefit of VEN addition to HMA by molecular ontogeny are supported by two recent risk models suggested for patients with AML treated with HMA + VEN, both included *TP53* as an adverse risk prognostic marker and secondary ontogeny was not considered adverse risk in either [24–26]. As most recommendations in the ELN guidelines are based on patients who were treated with intensive chemotherapy, our findings support the value of treatment-specific risk modeling, rather than “one model fits all” approach.

We note that the overall clinical outcomes observed in our study are worse than those reported in the VIALE-A trial (median OS of 9.9 vs. 14.7 months, respectively), reflecting the differences in patient characteristics between a routine care cohort and a clinical trial cohort. Although the age of patients in the DFCI cohort was similar to the VIALE-A trial, the disease and comorbidity profiles were different in substantive ways. First, in the DFCI cohort, 11% were exposed to prior HMA and 9% had prior alloHCT, whereas such patients were excluded from the VIALE-A trial. Second, 28% of the patients in our cohort had treatment-related AML which is known to be associated with *TP53* mutated AML [1, 27], as opposed to only 8% in the VIALE-A. Consequently, 35% of patients in the DFCI cohort had high-risk *TP53* mutated AML compared with 21% in the VIALE-A. In our sensitivity analysis excluding patients with a *TP53* mutation, the median OS of 14 months among patients treated with HMA + VEN was similar to the VIALE-A trial and reflects an association between worse outcomes and enrichment of *TP53* mutation.

The impact of alloHCT on long-term survival was demonstrated in the entire cohort and in each sub-group analysis, emphasizing the crucial role of transplantation in reaching a potential cure in patients with high-risk AML. Our results support the use of alloHCT in consolidating responses after HMA-based therapy in all transplant-eligible patients irrespective of their molecular ontogeny. Consistent with these results, other retrospective studies have shown encouraging results in patients consolidated with alloHCT after HMA + VEN, even when compared to patients treated with intensive chemotherapy upfront [28, 29]. With the expansion of alternative donor options and the development of improved conditioning and GVHD prophylaxis regimens [30–32], alloHCT post-HMA + VEN may become more accessible, even in an older population.

Our study has several limitations. First, its retrospective nature may cause potential selection bias; this was partially addressed by including all consecutive patients and using sensitivity analyses. Second, as in all real-world analyses, there is substantial heterogeneity between patients, which was addressed by stratification and regression analyses. Finally, we did not include measurable residual disease (MRD) data, which may aid in explaining the improved response and survival seen in secondary ontogeny with VEN. This will be a focus for future studies.

In conclusion, we demonstrate that in newly diagnosed AML patients treated with HMA + VEN, the definition of adverse risk defined by secondary mutations in the ELN 2022 risk criteria does not apply and survival is similar in patients with genetically defined secondary and de novo disease. This is a consequence of marked survival benefits in patients with secondary ontogeny treated with HMA + VEN. Conversely, in patients with *TP53* mutations, treatment with HMA + VEN did not improve any clinical outcomes compared with HMA, calling into question the role of the addition of VEN in *TP53*-mutated AML.

### Supplementary information

Supplemental material

## Data Availability

For original data please contact the corresponding author.
