# Supplementary material for: Molecular ontogeny underlies the benefit of adding venetoclax to hypomethylating agents in newly diagnosed AML patients
Source: Leukemia. 2024 Mar 27;38(7):1494–500. doi: 10.1038/s41375-024-02230-w (PMC11216982; doi:10.1038/s41375-024-02230-w)
Supplement: Supplementary file 1 — Supplemental material [file 41375_2024_2230_MOESM1_ESM.docx]

Molecular ontogeny in AML is both prognostic and predictive in patients treated with
HMA plus venetoclax

Shai Shimony^1,2^, Julia Keating^3^, Jacqueline S. Garcia^1^, Evan C. Chen^1^, Marlise R. Luskin^1^, Maximilian Stahl^1^, Donna S. Neuberg^3^ Daniel J. DeAngelo^1^, Richard M. Stone^1^, R. Coleman Lindsley^1^

^1^Division of Hematologic Neoplasia, Department of Medical Oncology, Dana-Farber Cancer Institute, Boston, MA;^2^Rabin Medical Center and Faculty of Medicine, Tel Aviv University, Tel-Aviv, Israel; ^3^Department of Data Science, Dana Farber Cancer Institute, Boston, MA

*Supplemental data*

Figures: 4

Tables: 5

Figures


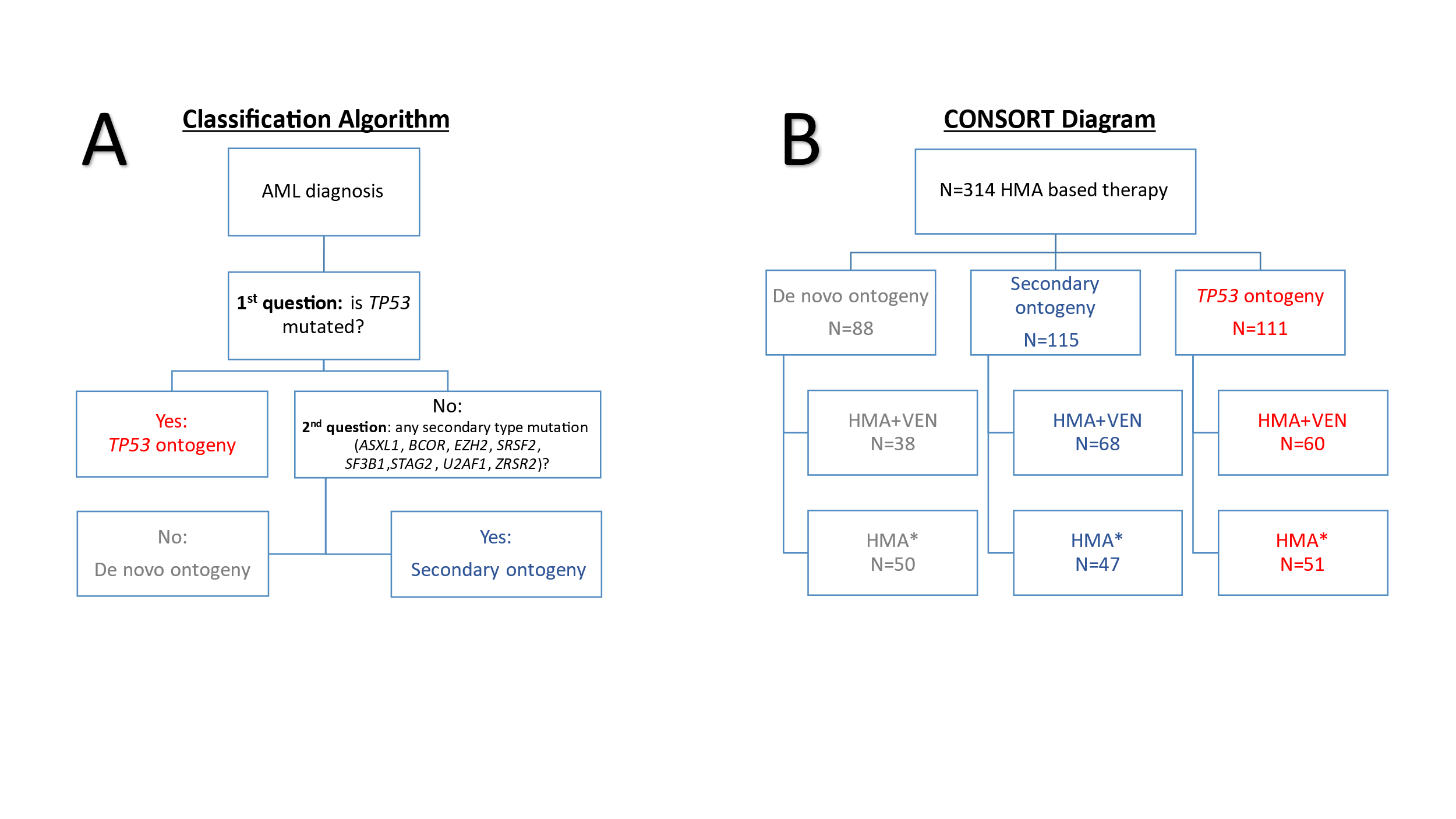


**Supplemental Figure 1. Cohort classification.
A. Molecular ontogeny classification algorithm. B. Consort diagram of the entire cohort.**

AML - acute myeloid leukemia; HMA - hypomethylating agent; VEN - venetoclax.
 *21/148 (14%) in the HMA group received additional non-venetoclax drug: FLT3 inhibitors (n=5), CD33 antibody (n=5), APR246 (n=3), Syk inhibitor (n=2), ipilimumab (n=2) and one of each of the following – CD123 antibody, CD47 antibody, IDH1 inhibitor, MUC1 inhibitor.


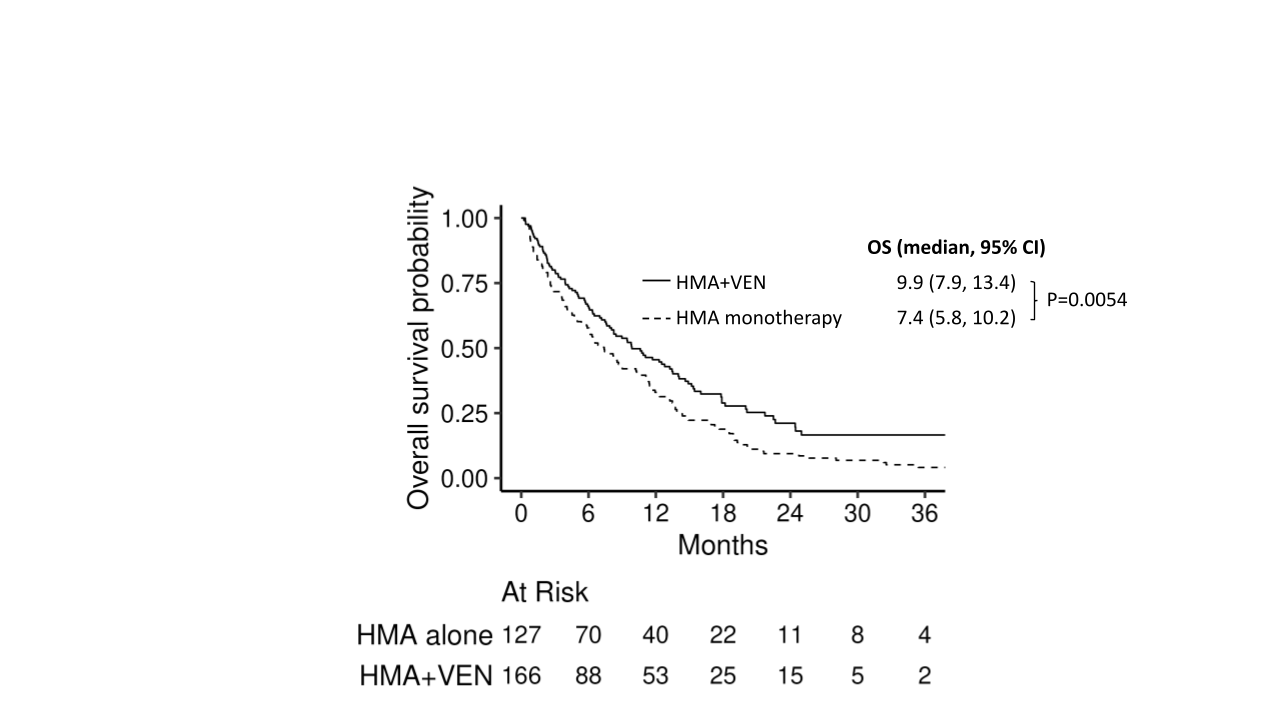


**Supplemental Figure 2.** **Overall survival of HMA+VEN vs. HMA excluding patients with non-VEN drug added to HMA.**OS – overall survival; CI – confidence interval; HMA – hypomethylating agents; VEN – venetoclax.


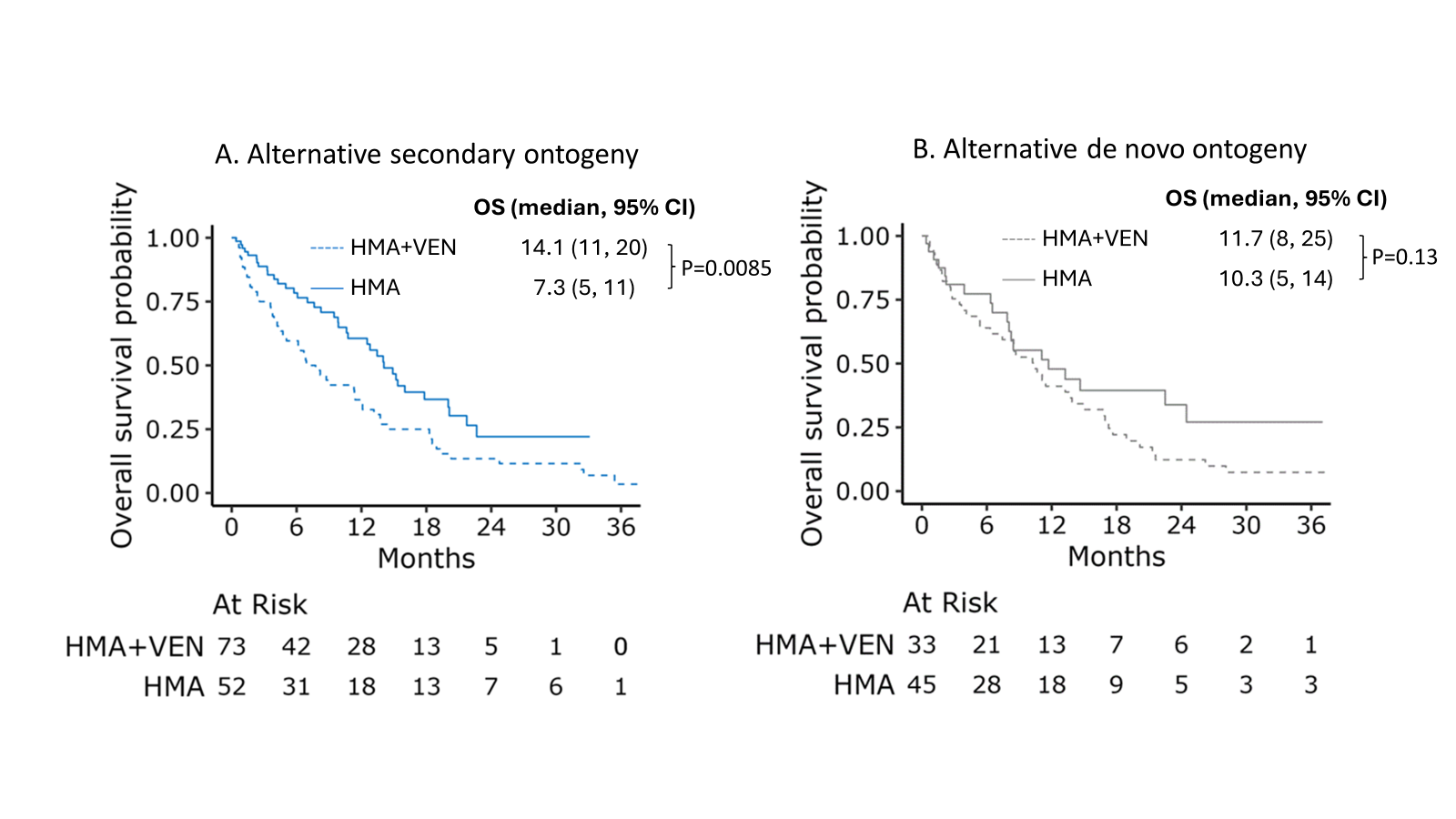


**Supplementary Figure 3**. **Overall survival in de novo and secondary ontogeny with *RUNX1* considered as secondary ontogeny-defining mutation.** Overall, 10 patients with *RUNX1* mutation and no other secondary ontogeny-defining mutation were reallocated from the de novo group to the secondary ontogeny group.

A. OS in Secondary ontogeny with *RUNX1* as an additional secondary ontogeny defining mutation.

B. OS in De novo ontogeny without *RUNX1*

OS – overall survival; CI - confidence interval; HMA – hypomethylating agents; VEN – venetoclax.

**
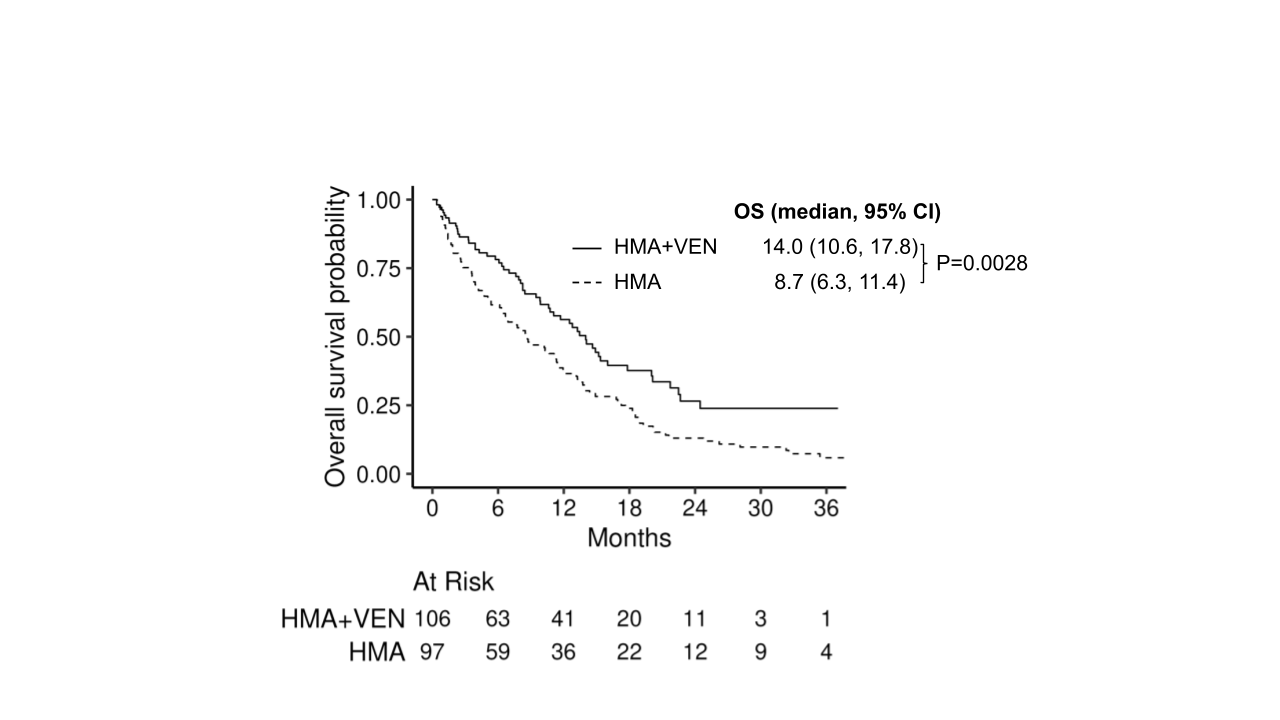
**

**Supplemental Figure 4. Overall survival of HMA+VEN vs. HMA excluding *TP53* ontogeny group.**

OS - overall survival; CI - confidence interval; HMA - hypomethylating agents; VEN - venetoclax.

Tables

|  | Overall (n,%) | HMA (n,%) | HMA+VEN (n,%) |
| --- | --- | --- | --- |
| Decitabine | 208 (66) | 109 (35) | 99 (32) |
| - 5 days | 166 (53) | 86 (27) | 80 (25) |
| - 10 days | 42 (13) | 23 (7) | 19 (6) |
| Azacytidine | 106 (34) | 39 (12) | 67 (21) |
| - 5 days | 17 (5) | 11 (4) | 6 (2) |
| - 7 days | 89 (28) | 28 (9) | 61 (19) |

**Supplemental Table 1. Hypomethylating agents type according to the treatment arm.**

HMA - hypomethylating agents; VEN - venetoclax

| Covariate | Univariable analysis (HR, CI 95%) | p-value | Multivariable analysis (HR, CI 95%) | p-value |
| --- | --- | --- | --- | --- |
| Age | 1.01 (0.99, 1.02) | 0.22 |  |  |
| Sex (relative to female) | 0.88 (0.68, 1.14) | 0.35 |  |  |
| Prior myeloid disease | 1.01 (0.77, 1.31) | 0.96 |  |  |
| Prior HMA exposure | 1.16 (0.79, 1.71) | 0.45 |  |  |
| Molecular |  |  |  |  |
| - Ontogeny group (relative to de novo) |  |  |  |  |
| - - Secondary | 1.04 (0.75, 1.44) | 0.81 | 1.12 (0.80, 1.56) | 0.50 |
| - - *TP53* | 1.65 (1.20, 2.27) | 0.0019 | 1.88 (1.37, 2.59) | <0.001 |
| - *FLT3-ITD* | 0.97 (0.61, 1.53) | 0.88 |  |  |
| - *NPM1* | 0.83 (0.56, 1.25) | 0.38 |  |  |
| - *IDH1* | 0.91 (0.53, 1.57) | 0.74 |  |  |
| - *IDH2* | 1.09 (0.73, 1.63) | 0.68 |  |  |
| - *NRAS/KRAS* | 1.30 (0.83, 2.03) | 0.26 |  |  |
| - *JAK2/CALR* | 1.35 (0.88, 2.06) | 0.17 |  |  |
| Cytogenetics (relative to normal)^1^ |  |  |  |  |
| - Complex | 1.62 (1.15, 2.28) | 0.0056 |  |  |
| - Others | 1.25 (0.86, 1.82) | 0.24 |  |  |
| Treatment |  |  |  |  |
| - HMA + VEN (vs. HMA) | 0.73 (0.57, 0.95) | 0.018 | 0.82 (0.63, 1.07) | 0.14 |
| - alloHCT as time varying covariate | 0.25 (0.14, 0.42) | <0.001 | 0.23 (0.14, 0.40) | <0.001 |

**Supplemental Table 2. Cox univariate regression analysis for OS in the entire group**

HR - hazard ratio; CI - confidence interval; OS - overall survival; HMA – hypomethylating agent; FLT3-ITD – fms-like tyrosine kinase 3 – internal tandem duplication; NPM1 – nucleophosmin 1; IDH – isocitrate dehydrogenase; NRAS – neuroblastoma rat sarcoma; KRAS – kirsten rat sarcoma; JAK2 – janus kinase 2; CALR- calreticulin; VEN – venetoclax; alloHCT – allogeneic hematopoietic stem cell transplantation.

^1^ 38 patients were omitted from the univariable regression analysis for normal cytogenetics and complex cytogenetics due to missing data.

|  | Median Overall survival (months), 95% CI | | |
| --- | --- | --- | --- |
| VAF group* | HMA | HMA+VEN | p-value |
| < 20%  (n=20) | 8.6 (1.0-21.6) | 5.7 (2.7-10.9) | 0.616 |
| 20% to < 50% (n=48) | 8.6 (4.1-17.0) | 5.9 (4.0-12.3) | 0.777 |
| 50% or more (n=43) | 4.96 (2.6-6.6) | 5.1 (1.9-9.0) | 0.278 |

**Supplemental Table 3. OS treatment comparison in *TP53* mutated AML stratified by VAF.**

CI - confidence interval; VAF - variant allele frequency; HMA - hypomethylating agents; VEN - venetoclax.

* There were only 9 patients with VAF < 10% (2 treated with HMA+VEN, 7 treated with HMA), thus survival comparison was limited due small sample.

| Covariate | Univariable analysis  (HR, CI 95%) | p-value | Multivariable analysis (HR, CI 95%) | p-value |
| --- | --- | --- | --- | --- |
| Age | 1.01 (0.99, 1.03) | 0.36 |  |  |
| Sex (relative to female) | 0.88 (0.63, 1.22) | 0.44 |  |  |
| Prior myeloid disease | 0.96 (0.68, 1.34) | 0.80 |  |  |
| Prior HMA exposure | 1.15 (0.72, 1.85) | 0.56 |  |  |
| Molecular | 1.05 (0.75, 1.45) | 0.79 |  |  |
| - Ontogeny group (relative to de novo) |  |  |  |  |
| - *FLT3-ITD* | 1.04 (0.63, 1.73) | 0.88 |  |  |
| - *NPM1* | 0.96 (0.63, 1.47) | 0.85 |  |  |
| - *IDH1* | 0.97 (0.49, 1.92) | 0.93 |  |  |
| - *IDH2* | 1.23 (0.79, 1.93) | 0.36 |  |  |
| - *NRAS/KRAS* | 1.40 (0.83, 2.36) | 0.21 |  |  |
| - *JAK2/CALR* | 1.38 (0.78, 2.44) | 0.27 |  |  |
| Cytogenetics (relative to normal) |  |  |  |  |
| - Complex | 1.46 (0.89, 2.40) | 0.13 |  |  |
| - Others | 1.25 (0.84, 1.85) | 0.27 |  |  |
| Treatment | 0.60 (0.43, 0.84) | 0.0031 | 0.66 (0.47, 0.92) | 0.015 |
| - HMA + VEN (vs. HMA) |  |  |  |  |
| - ALLOHCT as time-varying covariate | 0.25 (0.12, 0.49) | <0.001 | 0.27 (0.13, 0.53) | <0.001 |

**Supplemental Table 4. Cox regression univariable and multivariable analysis for OS in patients with de-novo or secondary ontogeny**HR - hazard ratio; CI - confidence intervalף OS - overall survival; HMA – hypomethylating agent; FLT3-ITD – fms-like tyrosine kinase 3 – internal tandem duplication; NPM1 – nucleophosmin 1; IDH – isocitrate dehydrogenase; NRAS – neuroblastoma rat sarcoma; KRAS – kirsten rat sarcoma; JAK2 – janus kinase 2; CALR- calreticulin; VEN – venetoclax; ALLOHCT – allogeneic stem cell transplantation.

^1^ 25 patients were omitted from the univariable regression analysis for normal cytogenetics and complex cytogenetics due to missing data

|  | Overall  (N=115) | Splicing mutations, N (%) | | p-value |
| --- | --- | --- | --- | --- |
|  |  | Splicing  (N=86) | Non-splicing  (N=29) |  |
| Sex (male) | 76 (66) | 58 (67) | 18 (62) | 0.7 |
| Age (median, range) | 74.4 (25.2, 89.6) | 75.2 (43.5, 89.6) | 73.1 (25.2, 88.2) | 0.2 |
| Age (% within age group) |  |  |  | 0.071 |
| - < 60 | 7 (6) | 3 (4) | 4 (14) |  |
| - 60 to <75 | 54 (47) | 39 (45) | 15 (52) |  |
| - ≥ 75 | 54 (47) | 44 (51) | 10 (34) |  |
| Prior MDS, MPN or MDS/MPN overlap | 51 (44) | 36 (42) | 15 (52) | 0.4 |
| Prior therapy |  |  |  |  |
| - Chemotherapy | 15 (13) | 11 (13) | 4 (14) | >0.9 |
| - Radiation | 17 (15) | 13 (15) | 4 (14) | >0.9 |
| - HMA | 17 (15) | 14 (16) | 3 (10) | 0.6 |
| - Prior AlloSCT | 15 (13) | 10 (12) | 5 (17) | 0.5 |
| Cytogenetics**^2^** |  |  |  |  |
| - Normal | 41 (41) | 30 (41) | 11 (41) | >0.9 |
| - 5/7/17 abnormalities | 14 (14) | 10 (14) | 4 (15) | >0.9 |
| - Complex karyotype | 13 (13) | 9 (12) | 4 (15) | 0.7 |
| ELN 2022 risk stratification |  |  |  | 0.2 |
| - Favorable | 14 (12) | 13 (15) | 1 (3) |  |
| - Intermediate | 2 (2) | 2 (2) | - |  |
| - Adverse | 99 (86) | 71 (83) | 28 (97) |  |
| First AML treatment |  |  |  | 0.7 |
| - HMA +/- non VEN drug^1^  o HMA monotherapy | 40 (35) | 31 (36) | 9 (31) |  |
| o HMA+ non-VEN drug | 7 (6) | 6 (7) | 1 (3) |  |
| - HMA+ Venetoclax | 68 (59) | 49 (57) | 19 (66) |  |
| Co-mutations |  |  |  |  |
| - *ASXL1* | 59 (51) | 36 (42) | 23 (79) | <0.001 |
| - *EZH2* | 7 (6) | 2 (2) | 5 (17) | 0.011 |
| - *BCOR* | 16 (14) | 10 (12) | 6 (21) | 0.2 |
| - *STAG2* | 16 (14) | 10 (12) | 6 (21) | 0.2 |
| - *NRAS* | 8 (7) | 4 (5) | 4 (14) | 0.11 |
| - *KRAS* | 7 (6) | 5 (6) | 2 (7) | >0.9 |
| - *IDH1* | 12 (10) | 9 (10) | 3 (10) | >0.9 |
| - *IDH2* | 19 (17) | 15 (17) | 4 (14) | 0.8 |
| - *NPM1* | 14 (12) | 14 (16) | - | 0.020 |
| - *FLT3-ITD* | 9 (8) | 6 (7) | 3 (10) | 0.7 |
| - *FLT3-TKD* | 2 (2) | 2 (2) | - | >0.9 |
| - *JAK2* | 8 (7) | 7 (8) | 1 (3) | 0.7 |
| - *PHF6* | 7 (6) | 7 (8) | - | 0.2 |
| - *SETBP1* | 9 (8) | 8 (9) | 1 (3) | 0.4 |

**Supplemental Table 5. Secondary ontogeny group characteristics by presence or absence of splicing mutations.** MDS – myelodysplastic syndrome; MPN – myeloproliferative syndrome; HMA – hypomethylating agents; alloSCT – allogeneic hematopoietic stem cell transplantation; CBF – core binding factor; ELN – European leukemia network; AML – acute myeloid leukemia; VEN – venetoclax. ^1^ 7/47 (15%) in the HMA group received additional non-venetoclax drug.
